# Study on Forming Mechanism of the Recast Layer on the Workpiece Surface during Micro EDM

**DOI:** 10.3390/ma17051073

**Published:** 2024-02-26

**Authors:** Chunmei Wang, Hao Wang, Xuyang Chu, Yunxiang Lu, Haifeng He

**Affiliations:** 1 School of Mechanical and Electrical Engineering, Guizhou Normal University, Guiyang 550025, China; 460099599@gznu.edu.cn (C.W.);; 2 Chongqing Shouxun Technology Co., Ltd., Chongqing 400066, China; 3 School of Aerospace Engineering, Xiamen University, Xiamen 361005, China

**Keywords:** micro EDM, recast layer, thermal influence, discharge energy, forming mechanism

## Abstract

In comparison to conventional EDM, micro EDM distinguishes itself through its brief discharge duration, narrow discharge channel radius, and concentrated energy density. However, there remains a paucity of comprehensive research on the surface formation characteristics in this domain. This paper delves into the formation mechanism of the recast layer in micro EDM workpieces, scrutinizing the primary factors that influence the formation process and the morphological attributes of the recast layer. We conducted a series of single-pulse experiments and micro EDM trials. Utilizing surface fitting tools, our experimental findings facilitated the derivation of a relational expression between the recast layer thickness of high-speed steel and the discharge parameters in micro EDM. Notably, when the energy is below 100 μJ, the recast layer thickness remains under 10 μm. Specifically, at an energy level of 16 μJ, opting for a smaller capacitance of 2200 pf and a higher voltage of 120 V in micro EDM results in a thinner recast layer. This study serves as a cornerstone for future efforts aimed at controlling and assessing the surface morphology of micro EDM.

## 1. Introduction

The EDM workpiece material suffers etching due to the thermal impact of single pulse energy. Consequently, the surface of the workpiece affected by thermal action consists of three parts: the solidification layer, thermal influence area, and matrix unchanged area [1].

On the outermost surface of the machined workpiece to be processed is the recast layer, which is mainly formed by the fusion of tool materials, workpiece materials, and elements of working liquid cracking. Because the thermal effect of EDM is the most serious, it has a serious impact on the characteristics and quality of the workpiece surface. During the heating stage of the workpiece material, the high temperature and high pressure of the plasma cause the kerosene medium to gasify and crack to form carbon atoms, and the carbon atoms form carbides with the metal atoms. At the same time, workpiece materials and tool materials that are affected by high temperatures are also melted and gasified, which results in the mixing of the process medium, tool material, and workpiece material. In the ejection stage of the electrical erosion product, the discharge channel generates a high instantaneous pressure due to the rapid expansion of plasma, forming an instantaneous pressure difference between the upper, lower, and outer layers. Later in the pulse discharge, the plasma channel breaks and the molten metal mixture under high pressure is pushed or ejected, mostly into the working medium. Under the action of surface tension and cohesion, the thrown metal material condenses into spherical particles with a small surface area, and the diameter of the spherical particle is about 0.5~100 μm [2]; under the cooling action of the processing medium, the remaining molten metal material is cooled and solidified rapidly, eventually forming a recast layer on the workpiece surface.

It is shown that the pulse width will also have a great impact on the recast layer thickness, especially under a small discharge energy, and a continuous and uniform recast layer can be obtained [3]. Lee et al. analyzed the relationship between recast layer thickness and electrical discharge machining parameters through extensive experimental research and found that peak current is the main factor affecting recast layer thickness [4]. Singh T. found that an appropriate servo reference voltage can reduce defects in the recast layer and improve surface quality [5]. S. Rao analyzed different combinations of EDM process parameters that are required to achieve higher MRR and lower SR for EN24 steel [6]. Tang et al. studied the recast layer thickness and heat affected layer. They found that the pulse duration has an important influence on recast layer thickness [7]. Ali et al. studied the effect of the total heat generated by an electrical discharge machine on white layer thickness (WLT) [8]. Liu J.F. found that more thermal damage can be found in EDM-processed surfaces including thick WL and HAZ and a thick martensite zone in particular by increasing the discharge duration and voltage [9]. H. Ramasawmy et al. studied the three-dimensional parameters of an EDM surface under different machining electrical parameters. They found from the experimental results that the pulse time and discharge current have an effect on the thickness of the recast layer, and they obtained the effect of peak current on the thickness of the recast layer and fitted the functional relationship of these parameters [1]. Syed et al. developed an eEmpirical model for WLT using response surface methodology (RSM) [10]. In order to minimize material removal, processing energy must be reduced to a very low level, so the power supply unit is an important component in micro EDM [11]. Fuzhu H. developed a new transistor-type isopulse generator for micro EDM [12]. Chu Xuyang et al. studied the mechanism based on two types of pulse generators in micro EDM using a single pulse discharge. These pulse generators provide a foundation for analyzing the forming mechanism of the recast layer on a micro EDM surface [13].

There are some differences in formation and morphology between micro EDM and common EDM [14,15]. In micro EDM, the surface morphologies are different under different discharge conditions, in which the single peak size and height of the recast surface can be best reflected [16]. In order to obtain high quality micro EDM, Singh A.K. et al. studied the optimization of micro EDM process parameters [17,18]. Although the single pulse discharge energy of micro EDM is small, the process of workpiece surface melting and recasting layer formation is short due to the small pulse width (usually less than 5 μs), and the morphology of the recasting layer is greatly affected by technological conditions. In this paper, starting from the single pulse recast layer, the formation mechanism of micro EDM is studied, the discharge parameters are analyzed, and a large amount of experimental research and analysis are carried out on the thickness of the stratum and recast layer.

As the discharge condition changes, the surface morphology shows different surface characteristics, such as drum bulge, flat pit, deep pit, etc. The single pulse discharge electrolytic process mainly consists of the workpiece material being heated to produce melting pool and molten pool etching; these two processes determine the morphology and surface characteristics of the recast layer. Therefore, it is of great significance to analyze the influence of these two processes on the formation of the whole recast layer and study the formation mechanism of the recast layer. Hence, the thermal mechanism of plasma and the ablation mechanism of molten materials are studied and analyzed.

Due to the use of special machining principles, micro electric discharge machining can easily control the minimum machining removal unit by changing the energy of small pulses, and theoretically, it can even achieve single-layer atomic level removal. In terms of the applicability of geometric machining, the micro electric discharge machining method can not only process flat and rotating bodies, but also process three-dimensional, complex, and free-form surfaces. Compared with MEMS technologies such as LIGA and lithography, the micro electrical discharge machining method has advantages such as simple equipment, strong implement ability, and three-dimensional machining capability. Meanwhile, this method processes a wide range of materials that are not only capable of processing various high-performance metals and alloys but also capable of processing semiconductor materials such as ceramics and silicon. These characteristics make micro electric discharge machining an irreplaceable method in the field of micro machining, and it has become a highly promising micro machining technology.

There has been limited research on the formation mechanism of micro electric discharge machining in the previous literature, and this article focuses on the study of the formation mechanism. By analyzing the forming mechanism of the recast layer, the related process parameters can be optimized to obtain a recast layer structure with less thickness or surface defects, improve the surface integrity of micro EDM, and expand its application in aerospace, MEMS systems, and other fields.

## 2. Analysis of Thermal Factors in the Forming Process of Recast Layer

**Figure 1:**
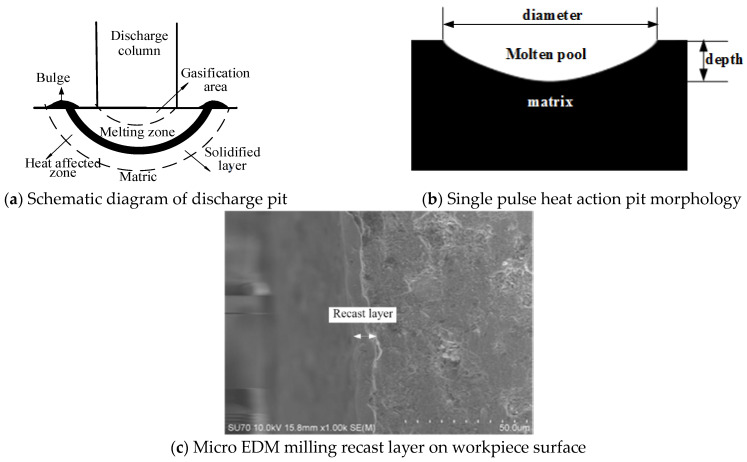
Recast layer morphology.

The melting pool on the workpiece surface is generated under the heat action of the plasma. In the process of single pulse discharge, the plasma rapidly heats the workpiece surface material to the melting point or even higher than the boiling point, forming a bowl-shaped melting pool, as shown in Figure 1b. During the erosion process of workpiece material, some of these materials will break away from the matrix with the bursting of bubbles and become the main part of the erosion of the workpiece material. The remaining molten material will be solidified on the surface of the workpiece. Figure 1a,c shows the morphology of recast layer. The size, depth, and temperature of the melt pool have an important influence on the forming morphology of the surface. The characteristics of the molted pool are related to the diameter, action time, and energy density of the plasma. A larger diameter of the plasma discharge channel, longer discharge time, and higher energy density can all increase the characteristic size of the molten pool. In a single discharge, the material stripping the substrate increases, resulting in a large crater diameter and depth on the workpiece surface. In single pulse micro EDM, the molten pool heating process is mainly affected by the parameters described in the following section.

### 2.1. Effects of Discharge Energy

According to the plasma expansion model and the plasma thermal reaction process, the size of the pit varies with different processing parameters due to the thermal effect of single pulse discharge.

(1) Discharge voltage, capacitance, and current

When discharge voltage, capacitance, and current increase, the expansion speed of the plasma radius increases [16]. The diameter and depth of pits affected by the monopulse process heat increase correspondingly, and the molten pool generated by the workpiece material heat is larger. Finally, the workpiece surface with superimposed large pits is formed under the action of continuous pulse.

(2) Discharge time *T*

When the pulse duration *T* is longer, the plasma discharge channel extends more fully, and the plasma radius is larger, so the heat-induced pit marks on the surface of the workpiece are larger. Moreover, with the increase in the discharge time, the formed melting pool is larger. This is because the heat conduction time of the workpiece material is longer, and the thermal effect of the melt in the pit is higher and more easily eroded.

### 2.2. Material Properties of Workpiece

In the process of micro EDM, the physical characteristics of the workpiece material have an important influence on the erosion of the workpiece material, and the different physical properties of the material lead to different degrees of thermal action in the process of micro EDM. When the specific heat of the workpiece material is larger, the metal material needs more heat energy when melting, and the diameter of the melt pool formed by a single pulse under the same energy is small, and the appearance of a single pit on the surface of the workpiece is also small. When the thermal conductivity of the material is small, the heat source will transfer less energy to other places during the heat effect, resulting in a smaller pit depth formed by a single pulse, less workpiece melting, and a thinner recast layer formed under the action of continuous pulses.

## 3. Analysis of Ablation Factors of Molten Material during the Forming of Recast Layer

**Figure 2:**
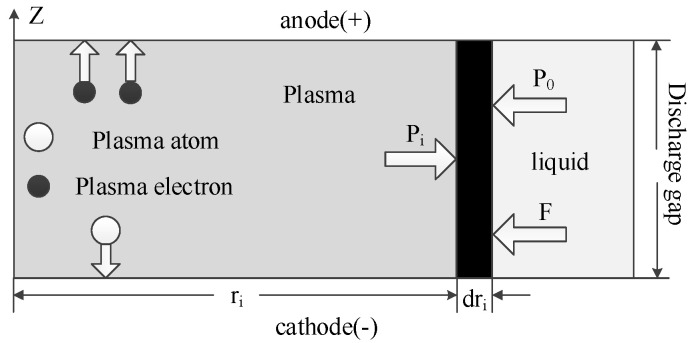
Force analysis diagram of plasma discharge channel [19].

During the ecliptic product-throwing phase of the single pulse discharge process of a micro-electric spark, the plasma is rapidly expanded by heat, and the discharge channel produces a high instantaneous pressure. The plasma constantly expands outward, forming an instantaneous pressure difference between the upper and lower levels and the inner and outer levels, as shown in Figure 2, where P_i_ is the pressure inside the plasma, P_0_ is the pressure of the processing medium on the plasma discharge channel, and F is the total accumulation of ions during the process. The moment the pulse discharge ends and the plasma channel bursts, the molten metal mixture is extruded out, most of it into the working medium to form small particles, and the other small part is squeezed into the hot pit around the channel, becoming part of the pit. When the energy of a single discharge is small, the pressure in the discharge channel is also small, resulting in a smaller impact when the bubble bursts, and less single spark corrosion. With the increase in the discharge energy, the pressure in the discharge channel increases correspondingly, resulting in a greater impact when the plasma discharge channel bursts, which increases the discharge erosion removal.

In the RC pulse discharge process, when the voltage and capacitance are large, the current in the plasma discharge channel increases correspondingly. When the voltage *U* and the current *I* in the discharge channel increase, the plasma temperature in the discharge channel is higher. When the temperature of the plasma increases, the pressure in the discharge channel also increases correspondingly, resulting in the plasma bubble rupture. The internal pressure has a more obvious influence on the melt heat, and the melt in the molten pool is more eroded. However, with the increase in discharge capacitance C, the single pulse discharge time is longer, resulting in the plasma bubble bursting at the end of the discharge. The final pressure in the discharge channel decreases, and the thermal shock effect on the melt decreases also to a certain extent.

In the RC pulse discharge machining, on the one hand, as the discharge capacitance and the discharge current increase, the size of the molten pool is large, and the temperature in the molten pool is also higher. On the other hand, with the increase in the discharge capacitance and discharge current, the final pressure in the discharge channel increases, resulting in a large thermal shock to the melt in the molten pool at the end of the single pulse discharge. The etching process is easier, and the amount of melt re-solidified on the workpiece surface is relatively small. Moreover, with the increase in the discharge capacitance C, the single pulse discharge time becomes longer, resulting in a relatively low pressure in the discharge channel when the plasma bubble bursts at the end of the discharge. However, due to the large heat transfer in the molten pool, the temperature of the molten material is relatively high, and it is very easy to discharge. Finally, under the action of continuous pulse, the surface recast layer presents a flat pit superposition phenomenon. When the discharge voltage *U* is low and the critical discharge gap is small, the molten material is difficult to etch, and more molten material is re-solidified on the workpiece surface. With the increases in the voltage and the increases in the critical discharge gap, the melt is more easily eroded and the solid re-solidification on the workpiece surface decreases. In addition, its energy efficiency decreases relative to the increase in the discharge gap.

**Figure 3:**
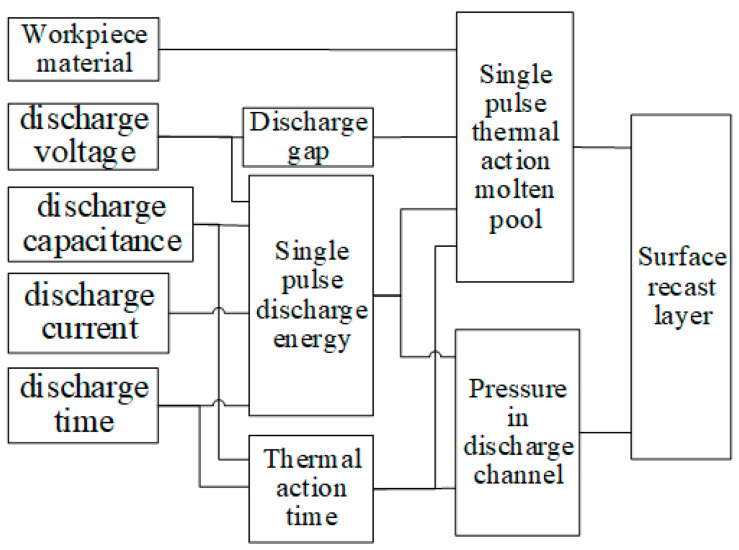
Model of the formation process of the recast layer.

Under different processing conditions, the forming process of the EDM workpiece surface is different. The relationship between various parameters and the recasting formation layer is shown in Figure 3. The quality of the recast layer on the surface of the workpiece is influenced by the morphology and size of the molten pool under a single pulse thermal action, as well as the pressure inside the discharge channel. The morphology of the single pulse thermal action melt pool is determined by the workpiece material, discharge gap, single pulse energy, and the time of thermal action. Meanwhile, the pressure inside the discharge channel depends on the energy of a single pulse and the time of thermal action.

## 4. Experiments

**Figure 4:**
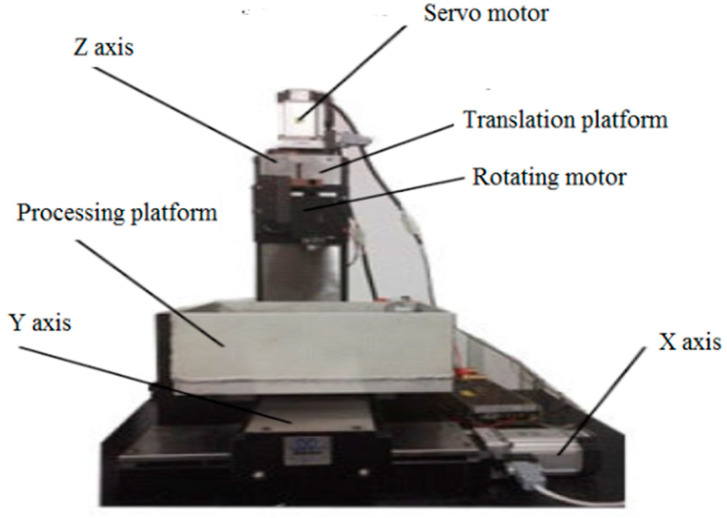
Three-axis motion control platform based on PMAC.

Based on the above mechanism analysis, a series of experiments with single pulse discharge were performed to investigate the different diameters and depths of the crater between the two types of pulse generators. Discharge voltages 45 V, 60 V, 90 V, and 120 V and discharge capacities 2200 pF, 4000 pF, 8800 pF, and 16,000 pF were applied in the experimental tests. Brass was used as a processing electrode. High-speed steel and TC4 titanium alloy were used as the workpiece material, and kerosene was used as the dielectric. The homemade micro EDM servo control system in Xiamen university can adapt to the machining characteristics of micro EDM and can achieve high-precision and high-efficiency micro machining. The three-axis motion control platform based on PMAC is illustrated in Figure 4.

**Figure 5:**
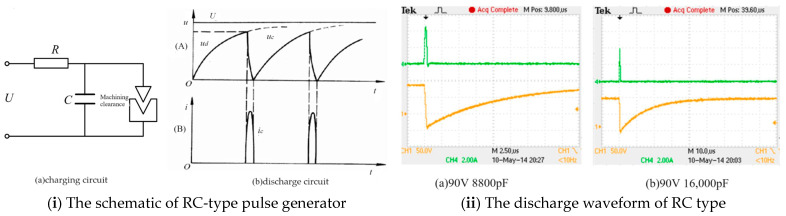
RC pulse discharge principle and discharge waveform diagram.

Single pulse discharge:

RC pulse power supply is used for processing, and the discharge principle is shown in Figure 5. The working principle of RC pulse power supply is shown in Figure 5, which consists of two circuits: one is the charging circuit, which is composed of DC power supply U, charging resistor R, and capacitor C; the other circuit is the discharge circuit, consisting of capacitor C, tool electrodes, workpiece, and the discharge gap between them. After the DC power supply is connected, the current is charged to capacitor C through the current-limiting resistor R. The voltage at both ends of capacitor C gradually increases exponentially. When the processing gap meets the breakdown condition, the energy stored on the capacitor is instantly released, forming a pulse current with a larger peak value. After the energy on the capacitor is released, the voltage drops to near zero, and the working fluid in the gap quickly returns to its insulation state. Afterwards, the capacitor is charged again, and the aforementioned process is repeated. During the RC pulse discharge process, discharge voltage and current are generated in the discharge gap. The voltage is collected using a Tektronix TPS2014 oscilloscope (Tektronix, Ltd., Johnston, IA, USA), and the current value is collected using a Tektronix TCPA300 current detection device (Tektronix, Ltd, Johnston, IA, USA). Figure 5ii shows the discharge waveform under different discharge capacitors at a discharge voltage of 90 V. When the discharge voltage is constant, the discharge current increases with the increase of discharge capacitance.

(1) Before the discharge experiment, the workpiece materials need to be polished. In the experiment, synthetic leather polishing pads and diamond grinding fluid were selected for polishing high-speed steel and titanium alloy.
(2) The discharge waveforms under each discharge condition were measured during the experiment. Tektronix TPS2014 oscilloscope (Tektronix, Ltd, Johnston, IA, USA) was used to collect voltage, and Tektronix TCPA300 current detection equipment (Tektronix, Ltd, Johnston, IA, USA) was used to collect current value. Thus, the discharge time, discharge current, and discharge voltage were obtained.
(3) After cleaning the machined parts, individual pits were scanned and inspected with a U70 SEM (Hitachi, Ltd, Tokyo, Japan) and SPA-400 scanning probe microscope (Seiko, Ltd, Tokyo, Japan) to obtain two-dimensional and three-dimensional morphological parameters of the pits.

The process of obtaining the recast layer on the surface of the formed workpiece:

(1) The workpiece materials are formed on a processing platform developed by Xiamen University. In this equipment, the precision of Z-axis reaches 0.1 μm. Accuracy of X axis and Y axis reach 1 μm. The travel of X, Y, and Z axes is 28 mm.

In the transistor pulse power supply, the pulse width range is 1 μs–1 ms, processing voltage range 0–150 V, and current range 0–1 A.

(2) The machined workpiece is embedded in the self-made polishing jacket, and the side of the workpiece is polished. Rough polishing was first performed on fine sandpaper, and then fine polishing and grinding were performed on synthetic leather polishing pad μM with 0–1 grain size sandpaper.
(3) In order to observe the difference between the recast layer and the substrate on the surface of the workpiece, the polished workpiece needs to be corroded. High-speed steel EDM workpiece is corroded by a mixture of HNO_3_ and industrial pure alcohol, with the ratio of HNO_3_ to alcohol being 4:96. Titanium alloy itself is a corrosion-resistant alloy metal. The mixture of hydrofluoric acid, nitric acid, and distilled water is selected to corrode the titanium alloy EDM workpiece. The ratio of HF/HNO_3_/H_2_O is 1:2:7, and the corrosion time is 20 s.
(4) Before testing the workpiece with a scanning electron microscope, it is necessary to clean the workpiece. Put the processed workpiece into a container containing acetone solution, clean it with an ultrasonic cleaner for about 30 min, and then take it out to dry.
(5) After drying, the workpiece can be inspected by scanning electron microscope. In this paper, SU-70 scanning electron microscope (Hitachi, Ltd, Tokyo, Japan) is selected.

## 5. Results and Discussion

### 5.1. Recasting Layer Forming

**Figure 6:**
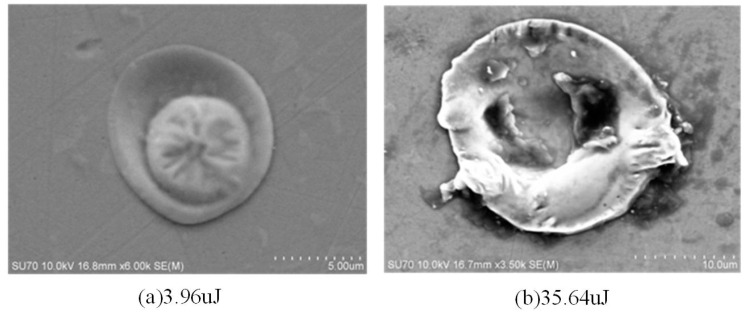
Single pulse discharge pit morphology with different energy.

**Figure 7:**
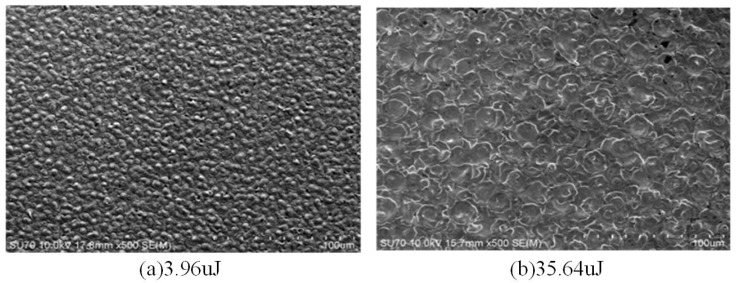
Continuous discharge workpiece surface topography with different energy.

In the single pulse machining process, if the single pulse discharge energy is small, the single discharge erosion generated by the single pulse discharge is lower, and the molten material in the thermal action pit solidifies into small protrusions under the cooling of the discharge medium, as shown in Figure 6a. Table 1 shows the pit diameter of the monopulse discharge experiment. When the energy is 3.96 μJ, the pit diameter is 10.23 μm and the pit shape is a small bulge. When the single pulse discharge energy is large, the diameter and depth of the molten pool increase, the melt increases, and the discharge trace is in the shape of a crater. The partially molten metal re-solidifies around the pit, as shown in Figure 6b. When the energy is 115.2 μJ, the pit diameter is 27.97 μm, and the pit shape is a plane. In the case of the same energy, the larger the capacitance, the larger the pit diameter, indicating that discharge capacitance has a greater impact on EDM, followed by discharge voltage. Under continuous machining conditions, flat pits are superimposed on the surface topography, as shown in Figure 7. The variation in recast surface morphology with discharge parameters is shown in Table 2.

**Figure 8:**
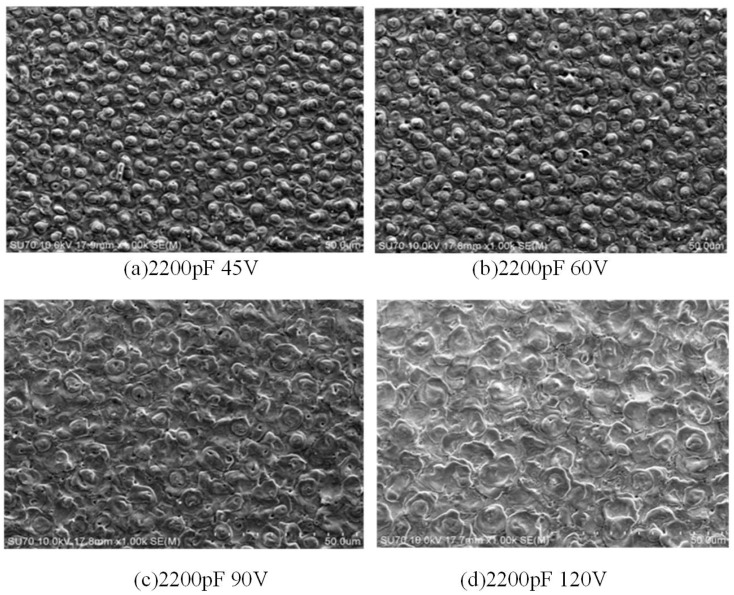
The surface morphology at different voltages.

**Figure 9:**
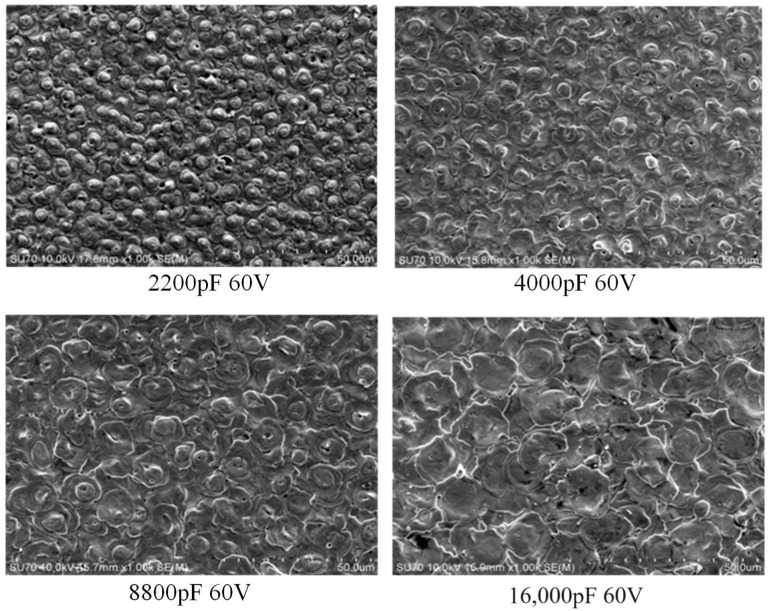
The surface morphology at different capacitance.

The surface morphology varies significantly with discharge capacitance and discharge voltage when RC pulse power is used for micro-machining. Figure 8 and Figure 9 show SEM images of the surface morphology. With the change in discharge parameters, the shape of the recast layer is different from that of the peaks on the machined surface, and the diameter of a single “hill” increases with the increase of discharge voltage.

(1) Effect of voltage on the formation of recast layer

**Figure 10:**
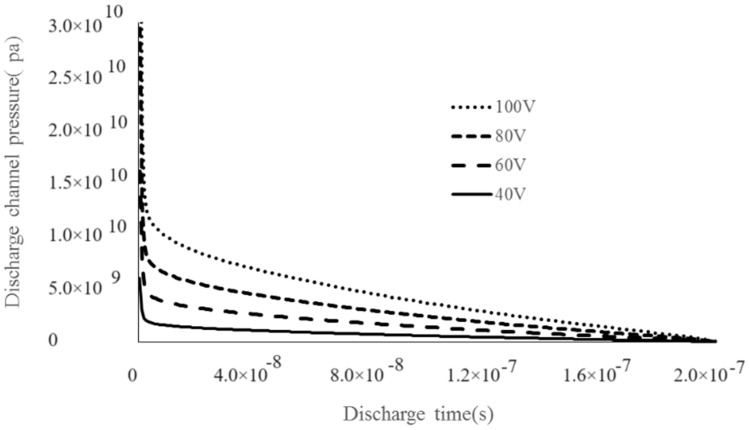
Variation law of discharge channel pressure under different discharge voltage (2200 pF) [19].

As shown in Figure 8, when the discharge capacitance is constant and the discharge voltage is low, the surface topography is formed by the overlapping of several small processes. With the increase in discharge voltage, the surface morphology of the recast layer is composed of small flat pits, and the surface diameter of a single pit increases; the flatter the surface morphology, the greater the erosion amount [20]. According to the calculation of the plasma expansion model, the final pressure in the plasma discharge channel is small when the discharge voltage is low [19]. Figure 10 shows the variation in discharge channel pressure for a 2200 pF discharge capacitor with varying discharge parameters. According to the plasma expansion model calculation, when the discharge voltage is 45 V, the pressure in the discharge channel is small, the discharge gap is small, the molten metal is difficult to be etched, and the melt metal is re-solidified on the workpiece surface at a low pressure. The calculation of pressure in the plasma expansion model is helpful to theoretically analyze the formation process of the recast layer. Therefore, when the voltage is low, the workpiece surface will develop a small concave and convex “mountain” overlap phenomenon. With the increase in discharge voltage, the single pulse discharge energy increases gradually, and so does the discharge channel pressure. When the discharge voltage is 120 V, the pressure in the discharge channel is larger, and the impact force generated when the bubbles burst in the discharge channel increases, and the amount of discharge erosion is larger. Moreover, with the increase in voltage and the discharge gap, more fragments are eliminated, and the surface topography formed by continuous pulse discharge will appear as a large pit overlap phenomenon.

**Figure 11:**
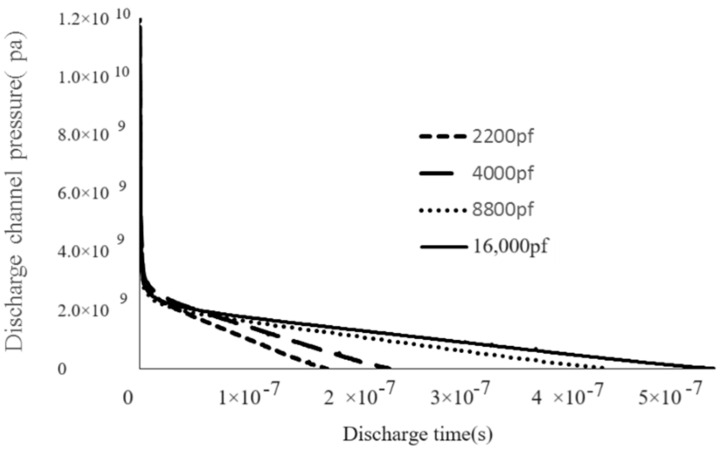
Variation law of discharge channel pressure under different discharge capacitance (60 V) [19].

In Figure 9, when the capacitance is small, the surface topography is formed by several small processes overlapping. With the increase in capacitance, the diameter of the single pit increases, and the surface morphology flattens, so the erosion amount increases. The surface morphology of the recast layer is composed of flat small pits, which is caused by the low pressure of the plasma discharge channel when the discharge capacitance is small. Figure 10 shows the variation in the discharge channel pressure with the discharge parameter at a discharge voltage of 60 V. When the capacitance is 2200 pF, the pressure of the discharge channel is small, the discharge energy is also small, and the molten metal is difficult to be etched, and finally it re-solidifies on the surface of the workpiece material at a relatively low pressure; therefore, when the capacitance is low, the surface of the workpiece exhibits a small bump “mountain pack” overlap phenomenon. As the discharge capacitance increases, the single pulse discharge energy gradually increases, and the discharge channel pressure also increases, as shown in Figure 11. When the discharge capacitance is 16,000 pF, the discharge channel pressure at the end of discharge is large, and the impact force generated by the discharge channel when the bubble bursts is large, and the discharge erosion amount is also large. Therefore, the surface morphology formed by the continuous pulse discharge exhibits a large pit overlap phenomenon, and the unetched melt forms a recast layer under the cooling action of the processing medium.

(2) Effect of discharge capacitance on the formation of recast layer

In conclusion, the discharge voltage and discharge capacitance directly affect the pressure in the discharge channel and the size of the discharge gap in micro EDM. With different processing parameters, the surface forming mechanism and surface morphology of the processed workpiece are different. When the discharge parameters are small, the pressure in the discharge channel is small, and the molten metal solidifies again under a very low pressure, forming from small protrusions. When the voltage and capacitance are large, the pressure in the discharge channel is large, the discharge erosion is also large, and the surface morphology will show a large pit overlap phenomenon.

### 5.2. Effect of Energy on Recast Layer Thickness

In order to study the thickness of the high-speed steel recast layer in RC pulse power processing, the machined workpiece is embedded in the self-made polishing jacket, and the side of the workpiece is polished. The polished workpiece needs to be corroded. It is necessary to clean and dry the workpiece. After drying, the workpiece can be inspected by scanning electron microscope. In this paper, an SU-70 scanning electron microscope is selected. When measuring the recast layer, it is necessary to take the average value after multiple measurements. A total of 16 processing parameters were designed: discharge voltages of 45 V, 60 V, 90 V, and 120 V; capacitance parameters of 2200 pF, 4000 pF, 8800 pF, and 16,000 pF.

**Figure 12:**
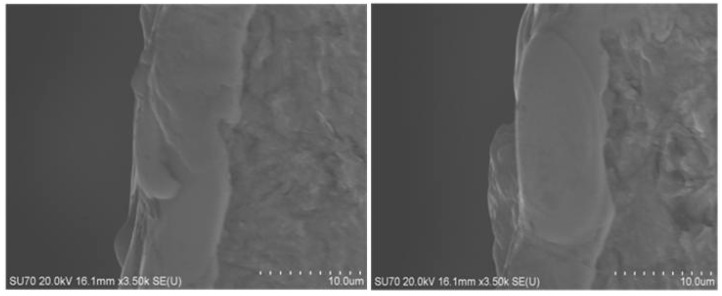
Thickness of recast layer at different places of the same workpiece under the same energy.

**Figure 13:**
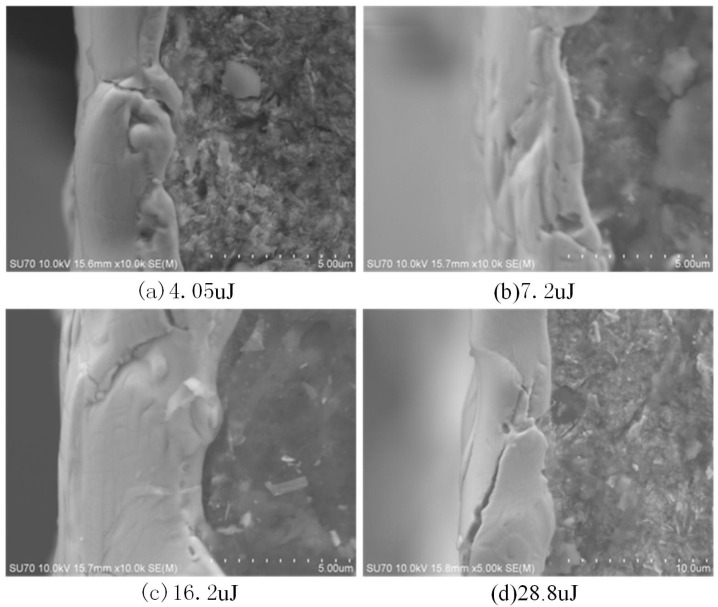
Scanning electron microscopy of recast layer thickness of high-speed steel under different energy levels of RC pulse power supply.

In micro EDM, pulse energy has a great influence on the recast layer on the workpiece surface. The recast layers of different thicknesses can be obtained with different pulse energy [21]. For micro EDM with RC pulse discharge power supply, the energy of a single pulse depends on the discharge capacitance and discharge voltage in the pulse circuit. Figure 12 shows the recast layer thickness at different parts of the same workpiece, and the machining energy of high-speed steel is 8.91 μJ. Under the same discharge conditions, the recast layer thickness on the surface of different parts of the workpiece is basically the same. Figure 13 shows the surface morphology of recast layer of high-speed steel under different discharge energy in RC pulse EDM.

The recast layer on the high-speed steel changes with the change in machining parameters. The recast layer thickness on the surface of EDM and machining parameters are shown in Table 3.

**Figure 14:**
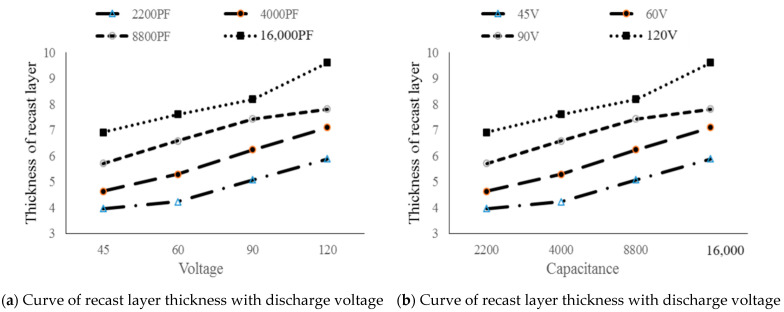
Variation curves of recast layer thickness of high-speed steel processed by RC pulse power supply.

In micro EDM, the recast layer thickness formed by the solidification of molten material on the workpiece surface increases with the increase in discharge energy. As shown in Figure 14a, when the capacitance is constant, the recast layer thickness increases with the increase in the discharge voltage. In Table 3, when the capacitance is 4000 pF, the voltage is 45 V and the recast layer thickness is 4.63 μm. When the voltage increases to 120 V, the recast layer thickness increases to 5.89 μm. As shown in Figure 14b, when the voltage is constant, the greater the discharge capacitance, the greater the recast layer thickness. When the discharge voltage is 90 V and the discharge capacitance is 2200 pF, the recast layer thickness is 5.08 μm. When the capacitance increases to 16,000 pF, the recast layer thickness is 8.91 μm.

In short, when the energy is less than 100 μJ, the recast layer thickness is less than 10 μm. Due to different processing conditions, it is difficult to obtain similar thickness morphology and consistency of recast layer thickness by various processing methods even if the same process parameters are adopted. In the ordinary EDM process, the thickness is 100 μm [1] due to its large processing parameters. During the machining process, the thickness of the recast layer is related to machining parameters, such as discharge parameters. For example, the machining medium can seriously affect the surface quality of EDM.

By surface fitting based on experimental results, the relationship between the recast layer thickness of high-speed steel material and capacitance and voltage during RC pulse power discharge machining in micro machining is fitted as follows:

(1) $$RLT=0.1568\cdot C^{0.2239}\cdot U^{0.4048}$$

where:

*RLT*—the recast layer thickness of high-speed steel during RC pulse power discharge machining, μm

*C*—discharge capacitance, pF

*U*—discharge voltage, V

**Figure 15:**
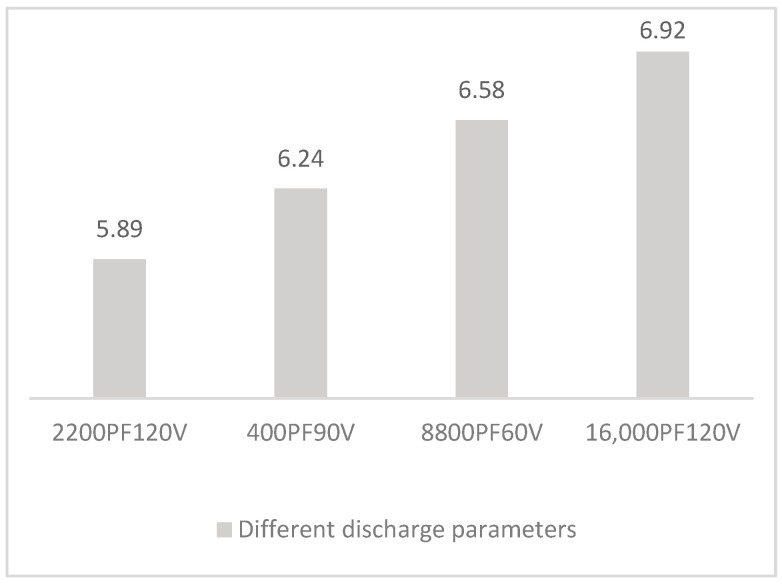
Comparison of recast layer thickness with different discharge parameters under the same energy on RC pulse discharge.

From Equation (1), discharge voltage and discharge capacitance have different effects on the recast layer during the discharge machining of RC pulse power supply, among which the increase in discharge voltage has a greater influence on the recast layer. When the voltage increases, the energy increases to a much greater degree than when the capacitance increases. With the increase in energy, the melting and gasification portions of the workpiece material and the re-solidification portion of the unpolished molten material on the workpiece surface increase correspondingly. As shown in Figure 15, when the energy is 16 μJ, the discharge parameters are 2200 pF and 120 V, 4000 pF and 90 V, 8800 pF and 60 V, and 16,000 pF and 45 V, respectively. The recast layer thickness is 5.89 μm, 6.24 μm, 6.58 μm, and 6.92 μm, respectively. Therefore, when the energy is 16 μJ in the machining process, the small capacitance of 2200 pF and the large voltage of 120 V are selected, and the thickness of the recasting layer is small. The larger the discharge capacitance, the longer the discharge time. Once the discharge time increases, the melt of the workpiece increases and the re-solidification of the material also increases, so the thickness of the recast layer increases correspondingly.

By studying the thickness of recast layer, it is found that machining parameters have a very important effect on the forming mechanism and surface morphology of the workpiece surface. With the increase in discharge voltage and capacitance, discharge current and discharge time also increase. The energy of the single pulse is also greater, and the melting and gasification portion of the workpiece material is correspondingly increased. The thickness of the recasting layer also increases with the increase in non-molten material on the workpiece surface.

### 5.3. Overall Characteristics of Surface Morphology of Different Materials

Under the same processing conditions, the surface morphology of different materials also varies. This mainly focuses on the analysis of the processing surfaces of titanium alloys, stainless steel, and hard alloys.

**Figure 16:**
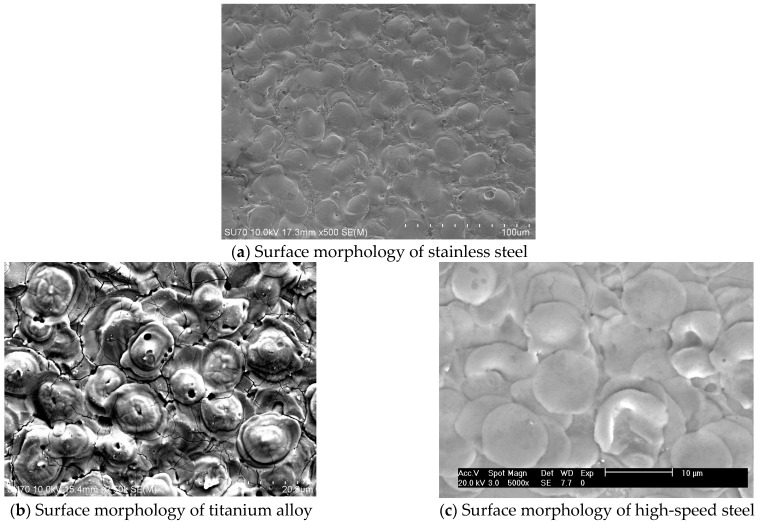
Surface morphology of different materials.

By scanning and detecting the micro electric discharge machining surfaces of different materials, it can be seen that the surface roughness and morphology of the machining surfaces of different materials are different, as shown in Figure 16. This is due to the different characteristics of materials, which have different physical parameters, resulting in different conduction processes and formation processes of molten materials.

For the micro electric discharge machining of stainless steel, the surface is uniformly distributed in flat and flat small pits, with obvious surface cracks and consistent recast layer thickness. The surface of titanium alloy processing shows a spherical distribution, which is related to the thermal properties of titanium alloy. The thermal properties of titanium alloy determine its surface spherical distribution and crack depth. The surface of high-speed steel machined workpieces is relatively smooth, with shallow depths of individual pits and basically no cracks on the surface. The surface quality is good and suitable for high-precision machining requirements.

## 6. Conclusions

The morphology of the recast layer on the surface of micro EDM was studied and the forming process of recast layer was described. The following conclusions are as follows:

(1) In micro EDM, the single pulse energy has great influence on the recast layer. The recast layers of different thicknesses can be obtained by different pulse energies. When the energy is less than 100 μJ, the recast layer thickness is less than 10 μm.
(2) When the pulse energy is low, specifically less than 10 μJ, the convex superposition of the recasting layer on the workpiece surface appears. When the pulse energy is larger than 10 μJ, the phenomenon of pitting superposition appears on the workpiece surface. During the machining process, the discharge voltage has an impact on the pressure within the discharge channel and the size of the discharge gap, ultimately affecting the morphology and size of the surface etching pit.
(3) In micro EDM, the recast layer thickness formed by the solidification of molten material on the surface increases as the discharge energy increases. The recast layer thickness can be reduced and the surface quality of machined parts can be improved by selecting machining parameters and strategies with smaller capacitance and higher voltage under the same energy. In the process of machining, the discharge capacitance affects the discharge time, thus affecting the workpiece material melt and the thickness of the recast layer.
(4) During the discharge machining process, the surface of the workpiece undergoes changes in its micro geometric and surface physical properties under the combined effects of heat, electricity, magnetism, and fluid dynamics. These changes manifest as changes in surface morphology, surface roughness, formation of surface metamorphic layers, and changes in surface mechanical properties. The surface morphology of different materials varies, and the surface roughness is also related to the material and the energy of electrical discharge machining. The surface roughness increases with the increase in electrical discharge energy.
(5) Compared with ordinary electric discharge machining, micro electric discharge machining has the characteristics of short discharge time, small discharge channel radius, and high energy density, so its discharge process is significantly different from ordinary electric discharge machining. Micro electric discharge machining is a novel breakdown mechanism based on the performance of magnetohydrodynamics and plasma, and the removal of materials depends on the combination of bubble mechanism and electronic mechanism. Micro electrical discharge machining has lower discharge energy, lower pressure in the discharge channel, and smaller discharge gap and discharge energy. Molten metal objects are difficult to corrode and will re-solidify on the surface of the workpiece material at relatively low pressure. Therefore, at low voltage, the workpiece surface will show a small convex “hill” overlapping phenomenon. As the discharge energy increases, the pressure inside the discharge channel is higher, resulting in an increase in the impact force generated when the bubbles in the discharge channel burst and a greater amount of discharge erosion. Moreover, with the increase in discharge voltage and discharge gap, more debris is removed, and the surface morphology formed by continuous pulse discharge will exhibit a large overlap of pits. The results of these single pulse experiments and continuous processing experiments are consistent with the research on plasma channel expansion studied in reference [21].

## Figures and Tables

**Table 1:** Pit diameter of single pulse discharge experiment.

Capacitance pF	Voltage V	Energy μJ	Diameter μm	Capacitance pF	Voltage V	Energy μJ	Diameter μm
2200	45	2.23	10.23	8800	45	8.91	16.13
2200	60	3.96	10.92	8800	60	15.84	20.72
2200	90	8.91	14.14	8800	90	35.64	22.38
2200	120	15.84	16.74	8800	120	63.36	24.06
4000	45	4.05	11.19	16,000	45	16.2	18.43
4000	60	7.2	13.12	16,000	60	28.8	21.78
4000	90	16.2	16.81	16,000	90	64.8	25.73
4000	120	28.8	20.28	16,000	120	115.2	27.97

**Table 2:** Recast layer surface morphology characteristics with different condition.

Key Parameter	Parameter Size	Recast Layer Morphology
Discharge current	small	Small bumps are superimposed
	big	Flat and flat pits are superimposed
Discharge capacitor	small	Small bumps are superimposed
	big	Flat and flat pits are superimposed
Discharge time	small	Small bumps are superimposed
	big	Flat and flat pits are superimposed
Material parameters	Lower heat conduction	The pit depth is small and the workpiece melts less
	When the specific heat is large	The diameter of the pool is small, small pit shape on the surface of the workpiece is small.

**Table 3:** Recast layer thickness of high-speed steel changing with discharge parameters during RC pulse power processing.

Capacitance pF	Voltage V	Energy μJ	Thickness of Recast Layer μm	Capacitance pF	Voltage V	Energy μJ	Thickness of Recast Layer μm
2200	45	2.2275	3.97	4000	45	4.05	4.63
2200	60	3.96	4.23	4000	60	7.2	5.3
2200	90	8.91	5.08	4000	90	16.2	6.24
2200	120	15.84	5.89	4000	120	28.8	7.11
8800	45	8.91	5.71	16,000	45	16.2	6.92
8800	60	15.84	6.58	16,000	60	28.8	7.61
8800	90	35.64	7.44	16,000	90	64.8	8.19
8800	120	63.36	7.82	16,000	120	115.2	9.61

## Data Availability

Raw data are available upon request.
